# Varicocoele. Classification and pitfalls

**DOI:** 10.1111/andr.13053

**Published:** 2021-06-08

**Authors:** Michele Bertolotto, Vito Cantisani, Francesco Maria Drudi, Francesco Lotti

**Affiliations:** ^1^ Department of Radiology University of Trieste, Ospedale di Cattinara Trieste Italy; ^2^ Department of Radiology University Sapienza of Rome, Policlinico Umberto I Roma Italy; ^3^ Andrology, Female Endocrinology and Gender Incongruence Unit, Department of Experimental and Clinical Biomedical Sciences “Mario Serio” University of Florence, Azienda Ospedaliero‐Universitaria Carreggi Firenze Italy

**Keywords:** classification, infertility, pitfalls, varicocele

## Abstract

**Background:**

Varicocoeles have been considered for a long time potentially correctable causes for male infertility, even though the correlation of this condition with infertility and sperm damage is still debated.

**Objective:**

To present a summary of the evidence evaluation for imaging varicocoeles, to underline the need for a standardized examination technique and for a unique classification, and to focus on pitfalls in image interpretation.

**Methods:**

Based on the evidence of the literature, the current role of ultrasound (US) imaging for varicocoeles has been reported and illustrated, with emphasis on examination technique, classification, and pitfalls.

**Results:**

US is the imaging modality of choice. It is widely used in Europe, while in other countries clinical classification of varicocoeles is considered sufficient to manage the patient. A number of US classifications exist for varicocoeles, in which the examinnation is performed in different ways.

**Discussion:**

An effort toward standardization is mandatory, since lack of standardization contributes to the confusion of the available literature, and has a negative impact on the understanding of the role itself of imaging in patients with varicocoeles.

**Conclusion:**

Use of the Sarteschi/Liguori classification for varicocoeles is recommended, since it is the most complete and widely used US scoring system available today.

Tubular extratesticular structures resembling varicocoeles, either at palpation or at US, should be identified and correctly characterized.

## 1 INTRODUCTION

Varicocoeles are abnormal dilatations of the pampiniform plexus with reflux of venous blood flow. It is present in 15% of the general male population, but it is more often identified in patients seeking medical attention for infertility.^1^, ^2^ This is why varicocoeles have been considered for a long time as potentially correctable causes for male infertility. However, a recent multicentric international study promoted by the European Academy of Andrology^3^, ^4^ reported in healthy, fertile men a prevalence of varicocoeles (∼37%) similar to that reported in primary infertile men.^5^, ^6^, ^7^ These data suggest that varicocoele may exert a scanty effect on male fertility, and that its surgical correction should be limited to highly selected populations. Accordingly, current EAU Guidelines on Male Infertility support nowadays very specific indications for varicocoele treatment both in adults and adolescents.^8^

Ultrasound (US) is the imaging modality of choice for varicocoeles.^8^ The body of published investigations is large, but exceedingly heterogeneous, and the role of imaging itself in the management of these patients is debated.^9^, ^10^ Outside Europe, US is not routinely used. Most important, both in and outside Europe US is performed in different ways, and several classifications are used.^2^

Recently, ESUR‐SPIWG ‐ the Scrotal and Penile Imaging Working group of the European Society of Urogenital Radiology ‐ released two papers to promote standardization of US for varicocoeles.^5^, ^6^ Recommendations are based on the evidence of the available literature and, when evidence is lacking, on best clinical practice and expert opinion. In these two papers, the most important features to consider when investigating a patient for varicocoeles are discussed, how to perform the US examination, and which classification is best.

### 1.1 Clinical classification of varicocoeles

Association between infertility, ipsilateral testicular atrophy, and varicocoeles regards clinically palpable, rather than non‐palpable disease.^11^ According to the criteria introduced in 1970 by Dubin and Amelar, varicocoeles are detected and scored clinically in three grades.^12^ Grade 1 varicocoele is palpable only while standing during Valsalva manoeuvre. Grade 2 is palpable also at rest while standing. Grade 3 is visible through the scrotal skin. Varicocoeles identified only at US (subclinical) are not considered. Some investigators suggest that clinical classification of varicocoeles is sufficient to manage the patient.^8^ Clinical scoring, however, is subjective, and depends significantly on the expertise of the sonologist. Also, the progression of subclinical varicocoeles to clinically evident disease is well documented,^13^, ^14^ and other pathologies can mimic varicocoeles at palpation.^5^ Based on these facts, there is a broad consensus among investigators that imaging plays a major role in the diagnosis of varicocoeles.^5^, ^6^

### 1.2 US classification of varicocoeles

**FIGURE 1:**
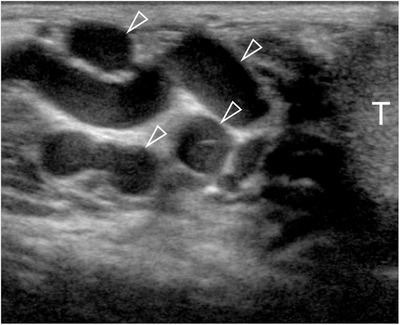
Identification of varicocoele at gray‐scale US. Serpiginous varicosities are seen (arrowheads) larger than 3 mm above the testis (T) with low‐level internal echoes

There is not a universally accepted system to classify varicocoeles. A number of classifications exist in which the exam is performed in different ways and a variety of parameters is evaluated^15^, ^16^, ^17^, ^18^, ^19^, ^20^, ^21^, ^22^, ^23^, ^24^ (Table 1). This fact has a negative impact on the understanding of the role of imaging in patients with varicocoeles, and contributes to the confusion of the available literature. An effort toward a standardization is mandatory. Both grey‐scale, color Doppler US and spectral analysis should be performed bilaterally, with the patient standing and supine, with and without Valsalva. Valuable information is obtained combining grey‐scale and Doppler interrogation. Once dilated veins around and/or above the testis are identified, key features to be evaluated are presence and characteristics of venous reflux, and testicular volume. According with ESUR‐SPIWG,^5^, ^6^ a maximum diameter ≥3 mm is considered diagnostic for a varicocoele (Figure 1). With the patient standing, during Valsalva manoeuvre, reflux > 2s is considered abnormal. Use of the Sarteschi/Liguori classification is recommended.^24^, ^25^ This is the most complete and widely used classification available today because the examination technique is clearly defined, and most of the parameters evaluated in the different classifications are included. In particular, characteristics of reflux are fully evaluated, as well as the position and site of the dilated veins and testis volume.

**TABLE 1:** Ultrasonographic classifications of varicocoeles

Study, year	Grades					Position
Sarteschi et al. (1993)	Grade 1: Inguinal reflux only during Valsalva in not enlarged vessels	Grade 2: Supra‐testicular varicosities with reflux only during Valsalva	Grade 3: Peri‐testicular reflux only during Valsalva in enlarged vessels. Visible but not dilated vessels when supine. Enlarged when standing	Grade 4: Enlarged vessels in supine and standing position, with increasing caliber during Valsalva. Reflux at rest, increasing during Valsalva. Possible testicular hypothrophy	Grade 5: Enlarged vessels in supine and standing position, with caliber not increasing with Valsalva. Reflux at rest, not increasing during Valsalva. Testicular hypothrophy. Intratesticular varices may be present	Standing & Supine
Hirsh et al. (1980)	Grade 1: No spontaneous reflux, inducible with Valsalva	Grade 2: Intermittent spontaneous reflux	Grade 3: Continuous spontaneous reflux			Standing
Dhabuwala et al (1989)	Grade 1: Reflux < 2s	Grade 2: Reflux > 2s	Grade 3: Spontaneous reflux increasing with Valsalva			Supine
Hoekstra & Witt (1995)	Grade 1: Dilated veins < 2.5 mm without flow reversal after Valsalva	Grade 2: Dilated veins 2.5‐3.5 mm and flow reversal after Valsalva	Grade 3: Dilated veins > 3.5 mm and flow reversal after Valsalva			Standing
Cornud et al. (1999)	Grade 1: Brief reflux < 1s	Grade 2: Intermediate reflux < 2s decreasing during and stopping prior to the end of Valsalva	Grade 3: Permanent reflux > 2s and with a plateau aspect throughout the abdominal strain			Not specified
Oyen (2002)	Grade 1: Slight reflux (< 2s) during Valsalva	Grade 2: Reflux (> 2s) during Valsalva, not continuous	Grade 3: Reflux at rest or continuous during the entire Valsalva maneuver			Supine
Pauroso (2011)	Grade 1: No varicosities seen. Reflux in the vessels of the inguinal canal that is observed only during Valsalva	Grade 2: Small varicosities with reflux seen only during Valsalva	Grade 3: Enlarged vessels whose caliber increases during Valsalva	Grade 4: Vessel enlargement with basal reflux that does not increase during Valsalva		Supine
Iosa & Lazzarini (2013)	Grade 1: Reflux > 1s only during Valsalva	Grade 2: Spontaneous, discontinuous reflux not increasing by Valsalva	Grade 3: Spontaneous, discontinuous reflux increased by Valsalva	Grade 4A: Spontaneous, continuous reflux not increased by Valsalva	Grade 4B: Spontaneous, continuous reflux increased by Valsalva	Standing & Supine
Patil et al. (2016)	Grade 0: Reflux time < 1s	Grade 1: Reflux time 1s‐2.5s	Grade 2: Reflux time 2.5s‐4s	Grade 3: Reflux time > 4s		Standing
Chiou (1997)	Maximum vein diameter (mm) 0: < 2.5 mm 1:2.5‐2.9 mm 2:3.0‐3.9 mm 3:≥4 mm	Plexus/sum of diameter of veins 0: No plexus identified 1: Plexus with sum diameter > 3 mm 2: Plexus with sum diameter 3–5.9 mm 3: Plexus with sum diameter ≥6 mm	Change of flow velocity on Valsalva maneuver 0: < 2 cm/s or duration n < 1s 1: 2–4.9 cm/s 2: 5–9.9 cm/s 3: ≥10 cm/s	Total score 0–9 ≥4: presence of varicocoele		Supine

**FIGURE 2:**
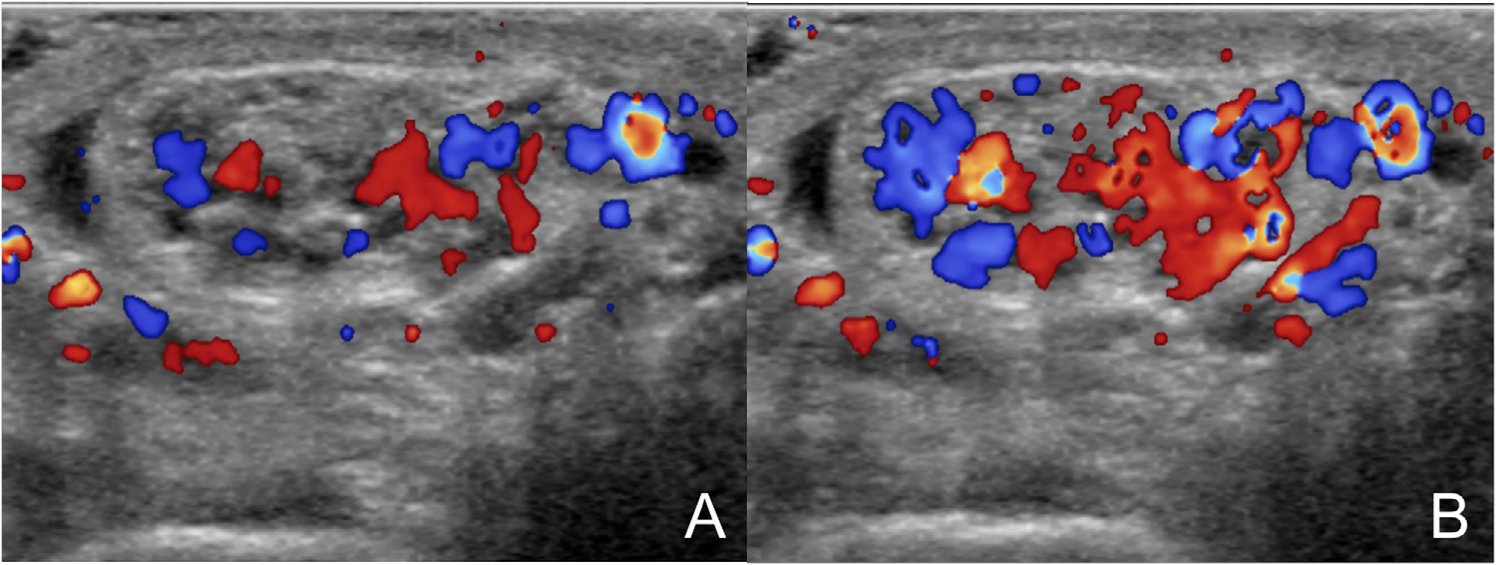
Grade 1 varicocoele according to the Sarteschi/Liguori scoring system. Images obtained at rest (A) and during Valsalva (B) showing inguinal reflux in non‐enlarging veins in standing position during Valsalva's manoeuver

**FIGURE 3:**
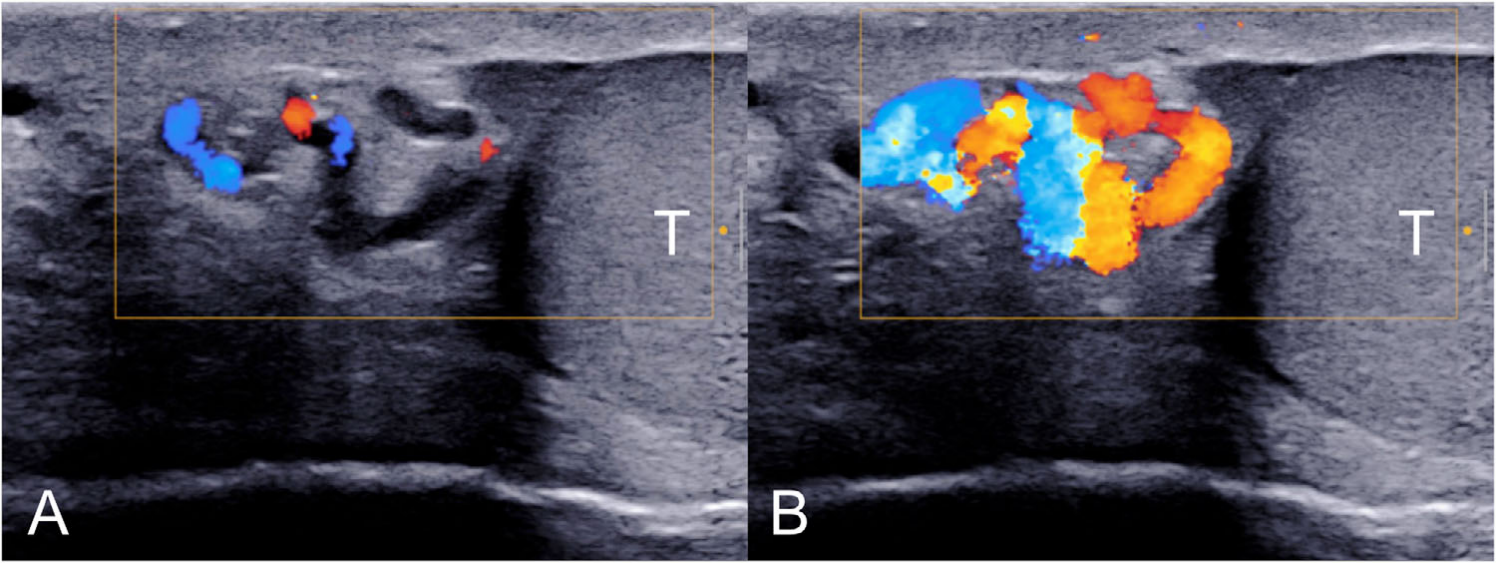
Grade 2 varicocoele according to the Sarteschi/Liguori scoring system. Images obtained at rest (A) and during Valsalva (B) showing reflux in supratesticular veins in standing position during Valsalva's manoeuver (T = testis)

**FIGURE 4:**
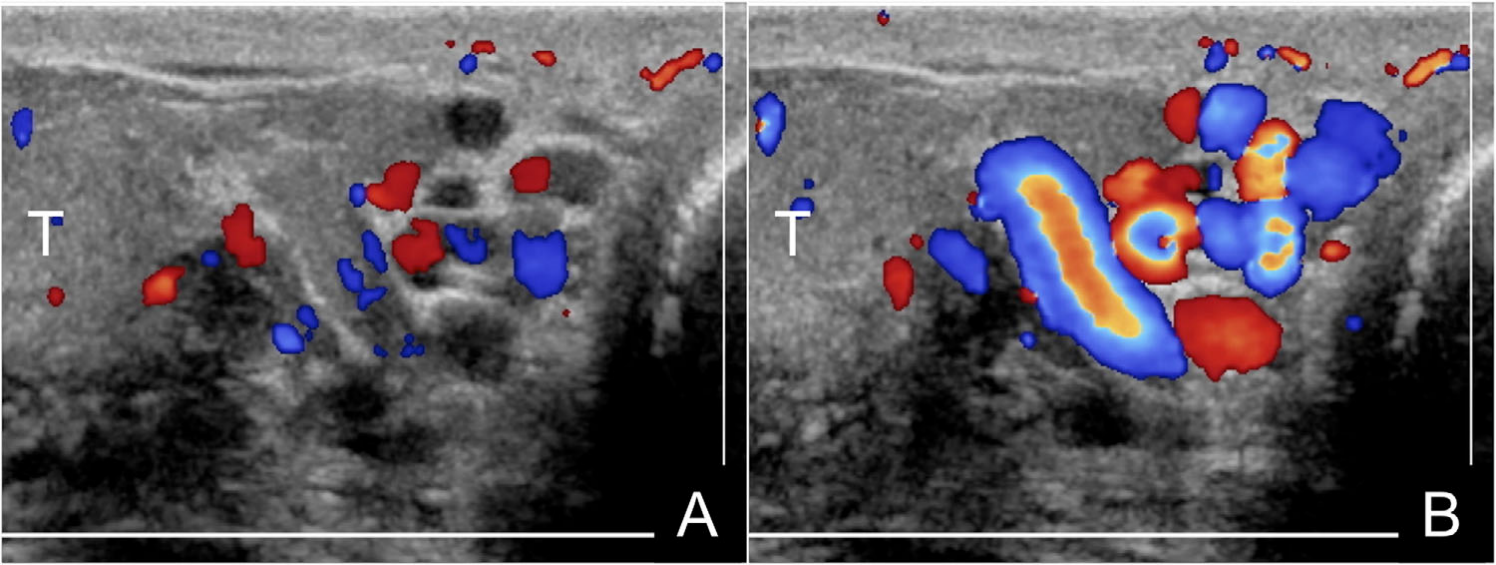
Grade 3 varicocoele according to the Sarteschi/Liguori scoring system. Images obtained at rest (A) and during Valsalva (B) showing reflux in the peritesticular veins in standing position during Valsalva's manoeuver (T = testis)

**FIGURE 5:**
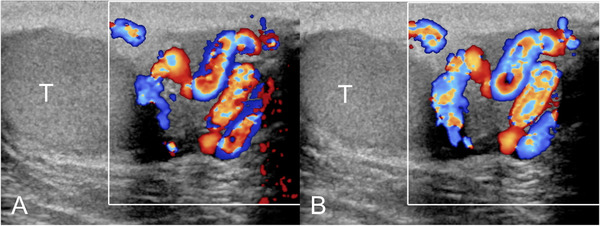
Grade 4 varicocoele according to the Sarteschi/Liguori scoring system. Images obtained at rest (A) and during Valsalva (B) showing reflux at rest in the peritesticular veins which increases during Valsalva's manoeuver (T = testis)

The Sarteschi/Liguori classification divides varicocoeles in five grades, depending on presence of varicosities, either in supine or standing position, and depending on the relationships of the dilated veins with the testis, testicular size, and characteristics of reflux. Grade 1 varicocoele is characterized by inguinal reflux in non‐enlarging vessels while standing during Valsalva manoeuvre (Figure 2). Grade 2 is characterized by varicosities with reflux only while standing during Valsalva that reach the superior pole of the testis (Figure 3). Grade 3 is characterized by varicosities also around the testis with reflux in standing position and during Valsalva maneuvre (Figure 4). Grade 4 is diagnosed if there are varicosities in supine and standing position which enlarge during Valsalva (Figure 5). Reflux is already present at rest and increases during Valsalva. Testicular hypotrophy may be present. Grade 5 is characterized by enlarged veins in supine and standing position. Reflux is already present at rest, and does not increase during Valsalva. Testicular hypotrophy is common.

Interestingly, the EAA US consortium defined “severe” varicocoele a venous vessel dilation (> 3 mm) characterized by a continuous venous reflux at rest, increasing or not during a Valsalva maneuvre, consistent with grade 4 and 5 varicocoeles according to Sarteschi/Liguori classification.^4^

### 1.3 How to perform US examination for varicocoeles

Gray‐scale, color Doppler, and spectral analysis have to be done. All parameters should be assessed bilaterally. The patient should be evaluated in both the supine and upright position, in general, upright position is more informative. This approach helps comparison among different studies and improves standardization, even though in clinical practice it might be unnecessary in some cases. Grey‐scale US is performed first. With the patient lying supine, enlarged veins are evaluated and testes volume are measured. The patient is then placed in standing position. The largest varicosity is identified and measured during the Valsalva maneuvre. However, measurement of the largest vein at rest is suggested by the EAA US consortium, to avoid the possible size variability due to Valsalva maneuvre.^4^ Colour Doppler and spectral analysis are then performed at the inguinal canal, in the supratesticular area, and in the veins around the testis.

### 1.4 Testicular volume

In a large series of healthy, fertile men a recent multicenter study reports a mean testicular volume of 20.4 ± 4.0 mL (measured with the Prader orchidometer). The 5th percentile of the testicular volume distribution is 15.0 and 14.0 mL for the right and the left testis, respectively.^4^

In varicocoeles, venous reflux is related with testicular hypotrophy, and repair can result in an increase of the testicular volume.^26^, ^27^, ^28^

In testis, volume is obtained more accurately from measurement of the three diameters at US rather than using an orchidometer, or with physical examination. Measurement of the testicular height (H), width (W), and length, (L) should be as accurate as possible. Testis compression should be avoided, since it influences significantly the measurements of the diameters. Estimation of the volume varies significantly using different mathematical formulas. The ellipsoid formula is widely used, also implemented in the US equipment for automated volume calculation from the three diameters. Testicular ellipsoid volume is obtained by multiplying the product of the three diameters by 0.52 (V = HxWxLx0.52). According to this formula, the 5^th^ percentile of the testicular volume distribution is 12.0 and 11.0 mL for the right and the left testis, respectively.^4^ Hence, testicular hypotrophy can be defined for volumes below these values. An empirical formula introduced by Lambert et al., has been shown more accurate than the ellipsoid formula.^29^, ^30^, ^31^ According to this formula, testicular volume is obtained by multiplying the three diameters by 0.71 (V = H×W×L×0.71). Lambert's formula is preferred by the ESUR‐SPIWG guidelines.^5^, ^6^ In a clinical setting, however, volumes calculated with the ellipsoid formula and measured using the Prader orchidometers fit better, while volume derived from Lambert's formula is larger. Hence, ellipsoid formula is preferred by the EAA.^4^ It must be underlined that volume calculated with the Lambert's formula is 27% larger than with the ellipsoid formula. Therefore, reporting the method used to calculate the volume is of paramount importance when imaging varicocoeles. It is possible to move from the volume obtained with the ellipsoid formula to Lambert's formula and the other way around multiplying by 1.36 and 0.73, respectively.

### 1.5 Presence, duration, and characteristics of reflux

The mainstay of the US examination for varicocoeles is Doppler evaluation of the duration of reflux. The therapeutic strategies for varicocoele correction are based on the assumption that the negative effect on spermatogenesis could reverse, if reflux is eliminated.^32^

Venous reflux is identified by combining color Doppler interrogation and spectral analysis.

**FIGURE 6:**
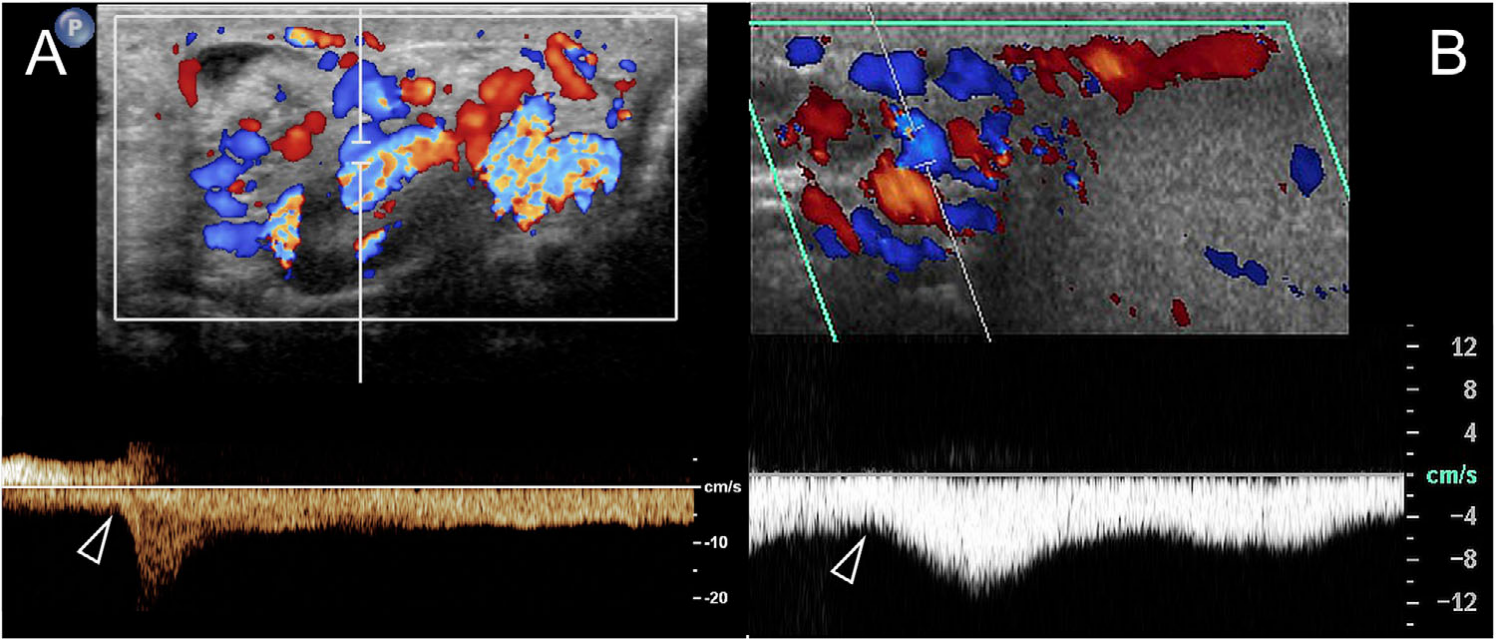
Waveform changes of varicocoeles in standing position during Valsalva manoeuver (arrowhead). (A) Inversion of reflux direction. (B) Increase of flow showing a plateau

Color Doppler interrogation of the spermatic vessels is panoramic. It is necessary to identify the varicosities and their relationship with testis. Moreover, it provides real‐time information on flow direction, and on how it changes in different positions and during the Valsalva maneuver. However, color Doppler assessment is subjective. Findings must be substantiated with spectral Doppler analysis which provides a measure of the duration and of the characteristics of reflux (Figure 6). The threshold fixed by the ESUR‐SPIWG guidelines for the diagnosis of varicocoeles is > 2s, measured in standing position during Valsalva.^5^, ^6^

### 1.6 Reflux peak velocity

Evaluation of reflux peak velocity is considered by several investigators a potentially useful Doppler parameter to predict the need for varicocoele repair.^33^ This is an active research field that might provide, in the future, important clinical information, but at present cannot be recommended for routine clinical use. Unfortunately, it is difficult to compare the results of the different available studies, since they differ in many critical points. Peak velocity is measured with the patient supine or while standing, either breathing normally, or during Valsalva. Measurements are performed in a variety of positions. Most important, in several investigations angle correction is not performed. The ESUR‐SPIWG does not recommend evaluation of reflux peak velocity in routine clinical practice because angle correction is essential in all Doppler velocity measurements, which also depend critically on the sampling site, patient position, and Valsalva.^5^, ^6^ Further studies obtained with a standardized examination technique are needed to substantiate the role of this parameter in the management of patients with varicocoeles.

### 1.7 Testicular and extratesticular abnormalities

In patients investigated for varicocoeles, a variety of atrophic parenchymal changes can be seen. Small, relatively hypoechoic testes with inhomogeneous echotexture or striated appearance can be identified by US.

Testicular hypotrophy can be secondary to high‐grade varicocoeles or, more often, an incidental finding due to prior cryptorchidism, infarction, infection/inflammation, or traumas.^34^, ^35^ Karyotype abnormalities should also be specifically considered, particularly Kleinfelter syndrome,^36^ showing hypergonadotropic hypogonadism. Hypogonadotropic hypogonadism should be checked too. It is important to identify testicular hypotrophy in infertile patients with varicocoeles since improvement of semen quality after repair is unlikely.

**FIGURE 7:**
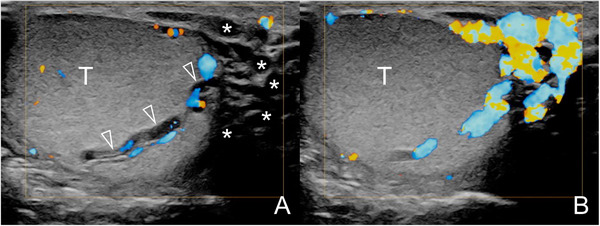
Intratesticular varicocoele associated with extratesticular varicocoele. Images obtained at rest (A) and during Valsalva's manoeuver (B). At rest (A) US reveals dilated intratesticular (arrowheads) and peritesticular (asterisks) veins with reflux during Valsalva manoeuver (B). (T = testis)

Intratesticular varicocoele can occur, either isolated or associated with extratesticular varicocoeles^37^ (Figure 7). US reveals dilated intratesticular veins with reflux during Valsalva manoeuvre. Small, nonpalpable testicular lesions can be discovered, whose nature cannot be assessed based on imaging and laboratory findings. Benign neoplasms and non‐neoplastic lesions are prevailing for nodules < 5 mm, making orchidectomy an inappropriately aggressive treatment. If tumour markers are negative, US surveillance is appropriate for the majority of testicular incidentalomas in infertile men.^38^

Extratesticular masses are often identified. Most of them are simple epididymal cysts, easily characterized by US.^37^ Solid and mixed nodules include a variety of neoplastic and non‐neoplastic lesions, the majority of which are benign. Differential diagnosis, however, is difficult.^39^

### 1.8 Reporting

Since in the various medical centers classification of varicocoeles may change, when comparing different US studies inconsistency of reporting is an issue. The correct evaluation of patients requires detailed description in the report of US and Doppler features. A standard report is welcome in which all the relevant features of the varicocoele are described. Regardless of the classification used, the following should be enclosed in the medical report: volume, echogenicity and echotexture of the testes; presence of varicosities and relationships to the testes; size of the largest vein measured while standing at rest (EAA standard operating procedures) and during the Valsalva maneuver (ESUR‐SPIWG operating procedures), irrespective of the location; characteristics of reflux before and during Valsalva, depending on the patient's position; incidental findings.^6^

### 1.9 Pitfalls

Tubular extratesticular structures resembling varicocoeles, either at palpation or at US, are often other pathologies. Spermatoceles, clusters of cyst, tubular ectasia, and other tubular structures such and post‐vasectomy changes are easily characterized at gray‐scale US.^37^ Cavernous haemangiomas may mimic a varicocoele on gray‐scale US. They show increased through‐transmission, heterogeneous echotexture, and enlarged vascular spaces that enhance at CEUS, but usually display no flows at Doppler interrogation, since velocities are too slow. Phleboliths may be seen as echogenic foci with distal acoustic shadowing.^39^, ^40^ Lymphangiomas may resemble haemangiomas at gray‐scale US, or present with cystic‐like appearance. The dilated lymphatics do not enhance at CEUS.^40^

**FIGURE 8:**
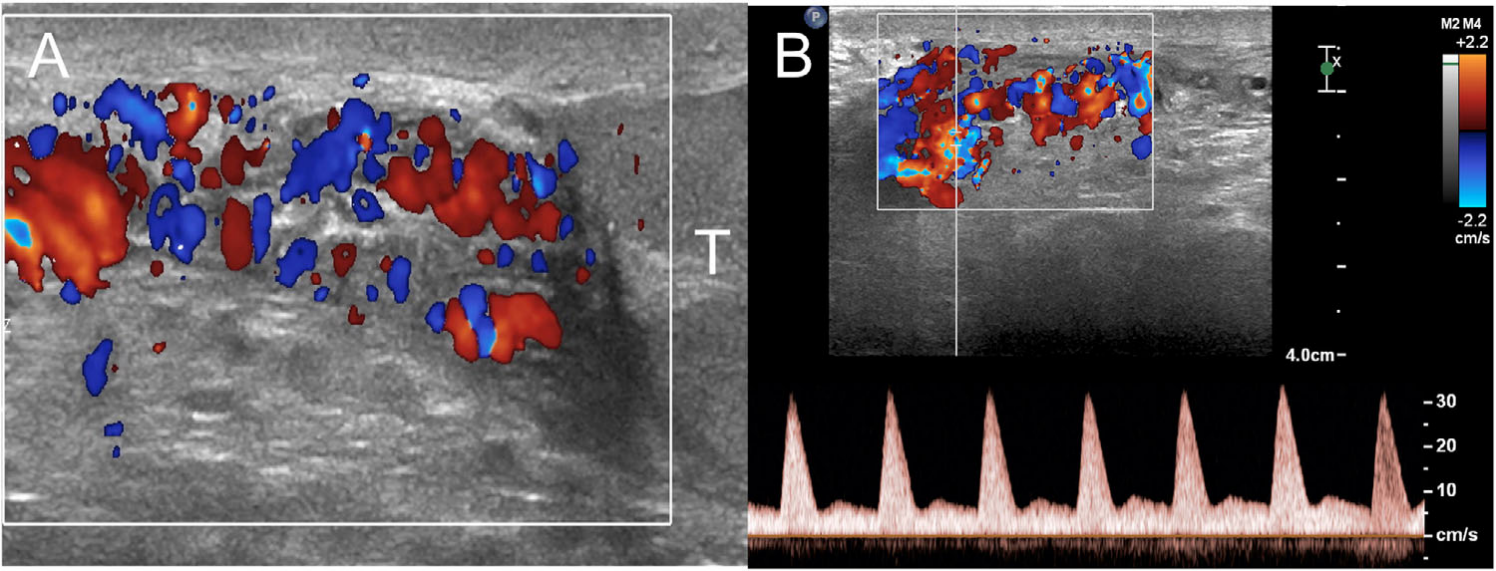
Scotal arteriovenous malformation mimicking varicocoele. (A) Colour Doppler US shows dilated vessels above the testis, resembling supratesticular varicocoele. (B) Spectral Doppler interrogation reveals high velocity arterial flows. (T = testis)

Arteriovenous malformations show large arteries with high velocity flows. This feature allows differentiation from varicocoeles, in which only venous flows are recorded^41^ (Figure 8).

Another mimic for varicocoele could be Zinner syndrome.^42^ The dilated vas deferens and epididymis can simulate venous dilatation, and during the Valsalva maneuver a Doppler signal resembling reflux can be artefactually recorded, due to spermatozoa movement.

**FIGURE 9:**
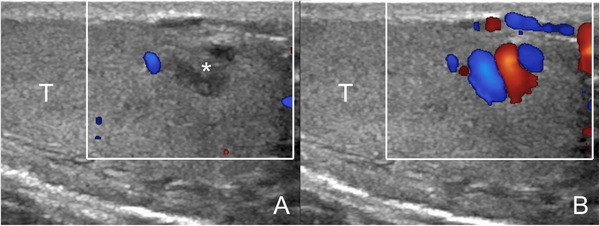
Intratesticular varicocoele. Images obtained at rest (A) and during Valsalva's manoeuver (B). At rest (A) a hypoechoic lesion is seen (asterisk) resembling a tumor. During Valsalva (B) enlarged intratesticular veins with reflux are revealed (T = testis)

Intratesticular varicocoeles can resemble lesions when investigated in the supine position at rest, but reveal their vascular nature when the patient is investigated in standing position during Valsalva manoeuver (Figure 9). Venous reflux is identified, a feature that allows differentiation with other vascular intratesticular lesions, such haemangiomas and arteriovenous malformations, which show arterial flows and arterialized‐venous spectral waveform.^5^

## 2 CONCLUSIONS

Although they are often asymptomatic and detected incidentally, varicocoeles are considered potentially correctable causes for male infertility. Diagnosis is obtained by US, but standardization is necessary, since there is no consensus on the diagnostic criteria, classification, and examination technique. The Sarteschi/Liguori classification is the most complete and widely used scoring system available today. Cysts, spermatoceles, tubular ectasia, post‐vasectomy changes, and other conditions which can mimic clinically varicocoeles are differentiated with multiparametric US.

## CONFLICT OF INTEREST

All authors declare that they have no conflict of interest

## AUTHOR CONTRIBUTIONS

Guarantors of integrity of entire study, MB, FL; study concepts/study design, all authors; manuscript drafting and revision for important intellectual content, all authors; approval of final version of submitted manuscript, all authors; literature research, MB, FL; manuscript editing, all authors.
